# Task-based profiles of language impairment and their relationship to cognitive dysfunction in Parkinson’s disease

**DOI:** 10.1371/journal.pone.0276218

**Published:** 2022-10-27

**Authors:** Anja Lowit, Tabea Thies, Julia Steffen, Franziska Scheele, Mandy Roheger, Elke Kalbe, Michael Barbe

**Affiliations:** 1 School of Psychological Sciences and Health, University of Strathclyde, Glasgow, United Kingdom; 2 Department of Phonetics, University of Cologne, Cologne, Germany; 3 Department of Neurology, University Hospital Cologne, Cologne, Germany; 4 Department of Medical Psychology, Neuropsychology and Gender Studies, University Hospital Cologne, Cologne, Germany; 5 Department of Psychology, University of Oldenburg, Oldenburg, Germany; Carl von Ossietzky Universitat Oldenburg, GERMANY

## Abstract

**Objective:**

Parkinson’s Disease (PD) is associated with both motor and non-motor problems, such as cognitive impairment. Particular focus in this area has been on the relationship between language impairment and decline in other cognitive functions, with the literature currently inconclusive on how the nature and degree of language impairment relate to cognition or other measures of disease severity. In addition, little information is available on how language problems identified in experimental task set-ups relate to competency in self-generated language paradigms such as picture description, monologues or conversations. This study aimed to inform clinical management of language impairment in PD by exploring (1) language performance across a range of experimental as well as self-generated language tasks, (2) how the relationship between these two aspects might be affected by the nature of the cognitive and language assessment; and (3) to what degree performance can be predicted across the language tasks.

**Methods:**

22 non-demented people with PD (PwPD) and 22 healthy control participants performed a range of cognitive and language tasks. Cognitive tasks included a screening assessment in addition to tests for set shifting, short term memory, attention, as well as letter and category fluency. Language was investigated in highly controlled grammar tasks as well as a Sentence Generation and a Narrative.

**Results:**

The study highlighted impaired ability in set-shifting and letter fluency in the executive function tasks, and a higher rate of grammatical and lexical errors across all language tasks in the PD group. The performance in the grammar task was linked to set shifting ability, but error rates in Sentence Generation and Narrative were independent of this. There was no relevant relationship between performances across the three language tasks.

**Conclusions:**

Our results suggest that there is a link between executive function and language performance, but that this is task dependent in non-demented PwPD. This has implications for the management of language impairment in PD, both for assessment and for designing effective interventions.

## Introduction

Parkinson’s Disease (PD) is a neurodegenerative disorder of the nervous system. Whilst PD has traditionally been regarded as a motor disorder [1–3], and is still diagnosed purely based on the presence of such symptoms [4], the prevalence of non-motor symptoms is increasingly being acknowledged. Amongst these, cognitive impairment is a widely recognised problem in people with Parkinson’s Disease (PwPD). A recent meta-analysis indicates that the pooled prevalence of mild cognitive impairment in PwPD is 40% [5] with about 1/5 of the patients already being affected at time of diagnosis [6, 7]. The cognitive dysfunction appears to particularly affect executive functions (which are mainly ascribed to dysfunction of dopaminergic fronto-striatal circuits) and memory, as well as attention, visuospatial functions and language impairment (ascribed to various neuropathological alterations, including limbic and cortical Lewy bodies, amyloid plaques, and cholinergic deficits) [6]. However, even though there is general agreement that cognitive impairment is a prominent feature of the overall symptom complex there is considerable disagreement in the literature on how the symptoms are related to each other and to other measures of disease progression, as well as how the various deficits progress over time [8].

One particular aspect of cognition with variable reports in the literature is the language domain. Many studies have highlighted impairments, both in relation to comprehension [9–11] and production of language [12–14]. Such impairments have been observed at the level of single word processing as well as sentence and discourse production, also referred to as complex language. Paradigms to assess single word processing have largely been experimental and observed deficits in naming ability [15–18], word generation [19–21], lexical decision tasks [22] as well as verbal fluency, with semantic fluency being generally more impaired than phonemic fluency [18, 23–26]. Performance differences have been identified in PwPD even at early stages, with processing and generating action verbs particularly impacted [27]. In relation to complex language, experimental set-ups range from highly controlled investigations of specific grammatical structures to task paradigms that generate more naturalistic language such as picture description, story retelling, spontaneous speech, etc. These studies have again highlighted a number of impairments in language comprehension and production, including decreased sentence comprehension [9, 10, 28, 29] as well as reductions in phrase length [30, 31], increases in pause number and duration [31–38], and a higher prevalence of mazes (false starts, repetitions, [37]) or dysfluencies (a measure combining mazes and pauses [13, 35, 39]). Furthermore, some PwPD have been shown to produce more grammatical errors [9, 13, 30, 32, 40], utterances with decreased syntactic complexity [31, 35] or reduced information content [31, 40, 41].

At the same time, there are studies that could not identify any significant differences between PwPD and healthy participants for the above measures [29, 39, 42, 43], and in some cases profiles diverged in the opposite direction, e.g. Garcia et al. [44] observed a higher rather than lower number of subordinate clauses in their PD group compared to healthy controls. Some of the observed variations can be attributed to differences in the severity of PD [45] or cognitive profiles of the participants [46]. Sample size is also likely to have affected the results, e.g. data in Murray and Lenz [43] indicate that at least some PwPD were performing below the normal range, but a small sample size of only 10 PwPD and 9 control participants rendered these differences non-significant.

Oher reasons for the observed differences could arise from task variations. For example, the various paradigms employed to assess complex language often differ in the level of constraint, i.e. participants are more limited in what utterances they can produce when generating sentences that have to include specific target words or describing a picture than in spontaneous discourse. This could either facilitate the task for them by providing some cues and limiting the choices, or it might make the task more difficult for them by requiring a specified output content or form that the participant might not have chosen themselves.

In addition to considering the constraint associated with a task, the degree to which different levels of conceptual and linguistic complexity can impact on performance should also be considered. The use of these two terms varies widely across the literature. For the purpose of this study, we adopted the definition provided by Palotti [47] who defines conceptual complexity as relating to how much information needs to be produced or processed. This can be manipulated through the nature of the material in a picture description task (information contained in it, level of assumed knowledge about events taking place, etc.), or target levels of information or information carrying units (ICUs) in a Sentence Generation task. For example, complexity increases from **“**The **man waves** to the **girl**” (3 ICUs) to **“**The **man waves** to the **girl** and the **boy**” (4 ICUs) and “The **man waves** to the **girl** but **not** the **boy”** (5 ICUs). Linguistic complexity, on the other hand, focuses on the degree to which a particular structure differs from what is considered the standard grammatical form of a language. For example, in English, past tense is usually formed by adding the morpheme “-ed” to the end of the word, and plural by using the suffix “-s”. Irregular past tense or plural forms, such as “went, felt, woke” and “children, sheep, foci” are thus linguistically more complex. Equally, grammatical constructions that deviate from the usual subject-verb-object structure, such as passive constructions, fall into this category. Other examples include relative clauses (“The **man** *who pushes the* ***pram*** waves to the **girl**”) which are linguistically more complex than coordinated structures such as “The **man** pushes the **pram** and waves to the **girl**” despite both of these sentences containing the same number of ICUs. Complexity can be further manipulated within relative clauses depending on their function (subject vs object relatives: “The man who pushes the horse” vs “The man who the horse pushes” and positioning within the main clause (embedded or final position: (“The man *who pushes the pram* waves to the girl” vs “The man waves to the girl *who pushes the pram*”).

Finally, tasks can differ in relation to how many stages of the language production process need to be completed, as reviewed by Altman and Troche [12]. For example, do they provide the linguistic material and only require manipulation of some grammatical feature, such as turning a sentence in present tense to past tense, or an active into a passive, or do they also require the participant to generate the content such as in a picture description or spontaneous speech task. Additionally, in the latter case it is important to consider to what degree the task is purely concerned with appropriate generation of lexical and grammatical content, or is requiring the participant to also take pragmatic choices into account to make their output relevant to an interlocutor etc. The more aspects need to be considered in generating the desired language output, the higher the processing costs of the task.

Whilst the tasks employed to date to assess language impairment in PD cover the full range of the above mentioned complexities, very few have actually purposefully compared tasks of different levels. The few exceptions include Troche and Altman [13] who manipulated the conceptual complexity of a sentence production task by including sentences with one or two events. They found that the fluency and grammaticality of utterances was significantly affected by linguistic complexity, although completeness, a measure whether all actors and actions were named appropriately, was unaffected. In addition, no study to date has formally studied the impact of other task variations such as constraint or processing cost on PwPD.

Further research is therefore necessary to establish task dependent performance profiles in PwPD. This is important not only from a theoretical point of view, but also for clinical purposes. Practitioners favour structured tests as they are quicker and more reliable to analyse, and frequently make clinical decisions on their basis. By nature, such tests involve complex and infrequent constructions. However, as there are currently few investigations that specifically focused on performance variations across different task paradigms, it is uncertain to what degree the results of these tests provide practitioners with an accurate description of their patient’s level of functioning. Whilst no one would expect performance on a particular grammatical feature such as passive constructions to fully capture the breadth of problems the speaker might experience in everyday conversation, clinicians would at least need to know whether they might reliably predict the severity level of language impairment across tasks. In addition, from a management point of view, they require information on whether interventions targeting specific grammatical structures can have beneficial effects for general communication effectiveness.

Given that language is a cognitive skill, and that both areas share the underlying neural mechanisms, investigations have focused on the question whether the observed language impairments are a core deficit in linguistic processing or related to other cognitive dysfunction. Most studies point towards the fact that the two areas are closely interconnected, and whilst many investigations only performed global measures of cognitive performance such as the Mini-Mental State Examination (MMSE, [48]), Montreal Cognitive Assessment (MoCA, [49]) or Dementia Rating Scale (DRS, [50]), they have reported better performance with regard to utterance length [43], sentence complexity [43], information content [40] in PwPD who score more highly on these tests. In addition, specific executive functions such as working memory [13, 40], attention [9, 12, 40], inhibition [10, 12, 13] and set shifting [10, 12, 14, 51] have been particularly highlighted as being able to predict performance in language tasks, particularly in the processing of complex grammatical structures such as relative clauses, passives etc. [8, 10, 51, 52], also see Altman and Troche [12] for a review of how cognitive functions can impact on specific language tasks. In addition, Murray [40] observed that utterances were longer and grammatically more complex in PD participants with better short-term memory and attention in a picture description task. Zanini et al. [53] found similar correlations between grammatical errors and set-shifting ability in a spontaneous speech task in bilingual speakers with PD, although interestingly only in their first language, whilst Troche and Altman [13] identified a link between grammaticality, completeness and fluency in a sentence generation task which were predicted by executive function scores as well as working memory in case of the latter two measures.

However, Altman and Troche [12] caution against the assumption that all language deficits observed in PwPD are somehow linked to accompanying cognitive deficit, highlighting the possibility that neural pathways more prominent to language processing could be affected independently. They provide evidence of this fact in their investigation involving a sentence generation task where the PwPD performed significantly more poorly than the healthy control speakers even when cognitive abilities, were controlled for [13]. Similarly, other studies also report the presence of language problems unrelated to or in the absence of cognitive impairment, e.g. Bocanegra et al. [15] observed impairment of action verb naming in participants deemed cognitively healthy based on their MoCA [49] results. Applying the same cognitive screening tool, Liu et al. [54] found that the aphasia quotient, a combined score from the Western Aphasia Battery [55] did not correlate with the participants’ cognitive results [49], and that the participants with and those without language impairment did not differ significantly in their cognitive scores. Taking the opposite approach, Lewis et al. [56] split their PwPD into those with and without cognitive impairment, as reflected by the DRS [50], and observed that both PwPD groups presented with language difficulties. Some of these results can again be explained by methodological factors. For example, both Dick et al. [42] and Small et al. [46] observed that lexico-semantic characteristics (word retrieval, information content) were much more susceptible to cognitive changes than syntactic aspects of language production. It is also important to note that with the exception of Troche and Altman [13], none of the above studies which observed linguistic impairments to be independent of cognitive performance investigated specific cognitive functions and instead only report more global cognitive scores derived from screening assessments. The question therefore remains whether the contradictory results on the relationship between cognitive functions and language were due to methodological issues or whether there are indeed language impairments that present independently of any cognitive deficit.

The uncertainty about the underlying mechanisms causing language impairment again has significant impact on the clinical management of these problems in PwPD as a clear understanding is fundamental to the development of effective intervention models. Language impairment as a core deficit would necessitate direct treatment with linguistically based exercises. Language deficits as a function of cognitive impairment would suggest effective treatment should address the latter, with an expectation of transferable benefits for language skills. A number of cognitive rehabilitation trials have investigated benefits at the single word level, albeit most in terms of verbal fluency (e.g. [57, 58]) rather than more functional work retrieval tasks (e.g.[59]). Only Altman et al. [60] have investigated wider communication parameters such as information content to date. It is therefore essential to conduct further research exploring the relationship between language and other cognitive skills, particularly looking at a wider range of measures and tasks to clarify to what degree methodological differences have impacted on findings.

This paper describes a clinically motivated, exploratory study that aimed to explore how methodological choices can impact on findings regarding cognitive and language impairment in PwPD in order to inform clinical assessment of language impairment and its potential treatment. We focused on three knowledge gaps identified in the literature above–(1) the impact of the choice of cognitive assessment (global screening versus specific executive function tests) on the relationship between cognition and language skills, (2) the impact of the nature and complexity of the language task on this relationship, and (3) the consistency of impairment profiles across the various language tasks. The corresponding research questions and hypotheses were as follows:

Research Question 1: Is there a difference between cognitively matched PwPD and healthy control speakers in relation to selected executive function (set shifting, working memory, attention and verbal fluency) and language tests? Whilst this question would not necessarily provide fundamental new knowledge to the field, it was important to establish to what degree our group of participants could be compared to previous reports in the wider literature. Accordingly, we predicted that group differences will be apparent across both executive function and language domains.

Research Question 2: Does the relationship between language and cognition differ depending on the nature of the cognitive assessment? We predicted that executive function tasks would be more likely to highlight a relationship between these two areas than global cognitive tests as most of the previous studies that did not identify a significant link only measured global cognition rather than specific executive functions (e.g. [15, 54, 56]).

Research Question 3: Does the relationship between language and executive function differ depending on the complexity of the language assessment? Previous evidence was less clear on the effect of language task type on the anticipated relationship between executive function and language. As we were investigating PwPD with relatively mild severity levels, we hypothesised that tasks associated with higher levels of linguistic and conceptual complexity would be most likely to identify a link.

Research Question 4: Can the severity and type of impairment in one language task predict those in others? As discussed above, structured and more naturalistic language tasks differ in relation to their language processing demands, and tap into different levels of the language production model. Although similar cognitive skills have been associated with the various stages of language production (message and functional) [12], there is likely to be a difference in processing cost that could cause a variation in task performance. Leaning on related research on motor speech difficulties which also reports impact of task type on performance, in some cases as a direct function of cognitive complexity of the task [32, 61–63], we hypothesised that the impairment profile will be individual to specific language tasks.

## Method

### Participants

We recruited 22 PwPD and 22 age, gender and education matched healthy control participants. Exclusion criteria included other illness that can affect cognitive or linguistic performance, history or presence of speech and language disorders besides those associated with PD, depression, dementia, uncorrected hearing or visual problems that could affect task performance. Table 1 provides information on participant demographics, as well as the results of a number of baseline tests and medical background information on the PwPD. All were native, monolingual speakers of German. Seven men and 15 women participated in each group, with a mean age of 66.6 (PwPD) and 66.9 years (controls). In each group, nine participants had received more than 12 years of education (university studies or equivalent), and 13 participants less (school education). The study was approved by the Ethics Committee of University Hospital Cologne. Testing for all participants was carried out in a quiet room at the University Hospital over a period of 2 months. Speech recordings were captured using an Edirol R-09 Digital Audio recorder.

**Table 1:** Participant profile.

	PwPD											Control Participants				
Participant	Age	Gender	Education	PANDA	SR	Intell	LED	Clinical Subtype	H & Y	UPDRS III	Disease Duration (years)	Participant	Age	Gender	Education	PANDA
**PD1**	62	M	H	26	1,2,6	8.0	727.5	akinetic-rigid	3	32	9	**CON1**	55	F	L	29
**PD2**	75	M	L	22	N	6.5	1505.8	akinetic-rigid	3	23	4	**CON2**	68	M	L	28
**PD3**	56	M	L	18	N	8.0	575	akinetic-rigid	2.5	17	3	**CON3**	75	F	L	29
**PD4**	71	M	L	21	2,5	9.0	505	tremor-dominant	2	25	7	**CON4**	80	M	L	30
**PD5**	57	M	H	27	N	9.0	626	tremor-dominant	1	15	2	**CON5**	74	F	L	23
**PD6**	54	M	L	17	1,2,5,6	7.5	210	equivalent	2	18	1	**CON6**	67	F	H	30
**PD7**	65	M	L	22	N	8.0	510	equivalent	2.5	35	5	**CON7**	55	M	H	27
**PD8**	56	M	L	23	N	9.0	209	tremor-dominant	1	25	6	**CON8**	64	M	H	29
**PD9**	67	F	L	18	1,4,5	9.0	524	akinetic-rigid	1	3	1	**CON9**	76	F	L	17
**PD10**	76	F	L	23	1,2	7.0	650	akinetic-rigid	4	36	7	**CON10**	53	M	L	22
**PD11**	61	M	H	26	1,3,5	8.5	475	equivalent	2.5	28	5	**CON11**	53	M	L	27
**PD12**	80	M	H	17	1,2,6	8.0	329.75	akinetic-rigid	3	30	9	**CON12**	76	M	L	16
**PD13**	76	M	L	15	N	7.5	157	akinetic-rigid	2	31	5	**CON13**	71	M	H	23
**PD14**	57	F	L	25	1,5,6	8.0	490	akinetic-rigid	1	7	9	**CON14**	64	F	L	20
**PD15**	62	M	H	26	N	8.0	1861.13	akinetic-rigid	4	22	10	**CON15**	72	F	H	26
**PD16**	67	F	L	23	N	8.0	765	akinetic-rigid	3	29	10	**CON16**	63	M	H	29
**PD17**	59	M	H	22	1,2	9.0	789	akinetic-rigid	1	8	1	**CON17**	60	M	L	17
**PD18**	73	M	H	26	1,3,4,5,6	9.0	260	akinetic-rigid	2	16	5	**CON18**	62	M	H	25
**PD19**	74	F	H	23	1,5	8.0	0	akinetic-rigid	3	28	14	**CON19**	50	M	H	15
**PD20**	73	F	L	25	1	9.0	300	tremor-dominant	3	48	3	**CON20**	79	M	L	29
**PD21**	71	M	L	17	N	7.0	630	akinetic-rigid	3	ND	ND	**CON21**	77	M	H	26
**PD22**	73	F	H	29	1,3,4,6	8.0	454	akinetic-rigid	2	20	5	**CON22**	78	M	L	21
*mean/count*	x = 66.6	M = 15	H = 9	x = 22.3	Y = 13	x = 8.14							x = 66.9	M = 15	H = 9	x = 24. 5
*SD/count*	SD = 8	F = 7	L = 13	SD = 3.9	N = 8	SD = 0.74							SD = 9.6	F = 7	L = 13	SD = 4.9

Abbreviations: Gender: M = male. F = female; Education level: low (L) = below 12 years (up to High School), high (H) = 12 years or more (further education); SR: self-reported language problems: 1 = word finding, 2 = sentence length, 3 = comprehension, 4 = turntaking, = sentence planning, 6 = train of thought; Intell: Intelligibility rating; LED: Levodopa Equivalent Dose [59]; H & Y: Hoehn and Yahr rating; UPDRS III: Unified Parkinson’s Disease Rating Scale, part III; x = mean, SD = standard deviation, N: no problem; ND: no data

### Materials and examination protocol

The University Hospital covers a wide geographical area and patients can incur several hours travelling time to attend. The protocol was therefore designed in a way that allowed all tests to be completed within a single assessment session. Participants were offered frequent breaks between tasks as well as refreshments. They were also reminded that they could leave the experiment if they were too fatigued to continue. Including these breaks, the sessions tended to last around two to two and a half hours and no participant asked for more than 10 minutes breaks indicating that the sessions were not putting too much strain on their capabilities. The assessments were arranged into three major groups:

Step 1: All PwPD were initially examined by a neurologist, which included an update of their medical history and scoring of the UPDRS III [64] as well as their Hoehn and Yahr score [65]. They were tested for at optimum medication phases; none experienced any off-periods during the assessment. Control participants did not undergo any medical examination.

Step 2: Both groups of participants were asked to complete two eligibility tests to exclude those with dementia or unmanaged depression. To screen for cognitive problems, the Parkinson neuropsychometric dementia assessment (PANDA [66], cut-off at 15 points) was administered. Similar to other dementia screening tools, the PANDA investigates a range of relevant cognitive domains with five subtests including a word paired associate learning task with immediate and delayed recall, an alternating semantic verbal fluency task, a visuospatial task in which half-masked squares with dot patterns are presented and the patient is expected to find the pattern which emerges on removing the mask, and a working memory and attention task in which rows of numbers presented in a random number have to be repeated in a systematic order. To screen for depression, we administered the German version of the Geriatric Depression Scale [67], cut-off at 9 points). Once eligibility was confirmed, demographic information, including their level of education, was collected. In addition, information was gathered on whether they perceived any problems with cognition, speech and language. For cognition, we applied a neuropsychological diagnostic questionnaire used by the University Hospital Cologne which focused on memory, attention, executive function, spatial orientation, mood, sleep and reading and writing ability. For speech and language, we enquired about whether participants had noticed changes or difficulties with speech (voice, volume and articulation), word finding, sentence construction (length and complexity), following a conversation or processing information, contributing to a conversation and turntaking, and whether they ever lost the train of thought during a conversation. Only the reports for language difficulties are reported here (see column SR in Table 1).

Step 3: Participants then completed three blocks of assessments, (1) Speech Assessment, (2) Cognitive Assessment, and (3) Language Assessment.

#### Block 1—Speech

Given the impact of medication on motor performance, the speech assessment was performed first in order to ensure that the participants’ motor state during this task was as close as possible to that scored in the UPDRS for comparison purposes. As stated above, none of the participants experienced any off-periods during the session. The speech assessment included a reading passage which was perceptually evaluated by three experienced listeners using a 9 point scale that rates intelligibility and listener effort [68]. We used the average of their scores to represent the severity of the speech impairment.

Blocks 2 and 3 were subsequently presented in randomised order, with further randomisation of tasks within each block. We had to limit the number of tests we could conduct in order to maintain a reasonable assessment load for the participants. The choice of cognitive and language tasks was therefore based on functions that were most commonly described in the literature within this field of study, and/or used in clinical assessment, resulting in the following test battery:

#### Block 2—Executive functions

We focused on tests assessing executive functions that have most commonly been associated with language problems in PwPD in the literature reviewed above, selecting tasks in line with our previous research on cognitive decline in PwPD [69]. This resulted in a neuropsychological test battery including set-shifting, working memory, attention, and verbal fluency.

1. Trail Making Test (TMT [70]): The TMT measures visual scanning, divided attention as well as cognitive flexibility. The difference score between subtests A and B (TMT B-A) was calculated to control for influences of motor difficulties associated with PD. The score reflects the Trail Making Contrast [71]).
2. Digit Span [72]: A backward digit span tasks was used to assess working memory skills. Raw scores were used for analysis as the standardised scores were too broadly categorised into percentiles to provide useful values for group comparison. This was possible as the two groups were closely matched for age.
3. Brief Test of Attention (BTA, [73]): As the name suggest, this test was used to investigate participant’s attention skills. Both numerical and letter spans were assessed and scored as normal [74]; as for digit span, the total raw score was used.
4. Regensburger Verbal Fluency Test [75]: This study used both variants–category fluency, generating food items (the more common animal category had already been tested as part of the PANDA) and letter fluency (words starting with the letter S). The number of correct times produced within one minute was scored, with no reduction in scores for errors or repetitions.

#### Block 3—Language skills

Based on the information available on different task complexities and demands, we built a set of distinct tasks that varied according to these dimensions whilst at the same focusing on structures that had previously been found to highlight differences between PwPD and healthy control speakers.

To allow us to investigate question 3, we constructed three grammar tasks that included linguistically complex constructions (passives and relative clauses) and were further controlled in terms of conceptual complexity. To answer question 4, we presented participants with additional language tasks that involved less constraint but higher processing costs. These included a Narrative Task (description of the Cookie Theft Picture [76]), which was associated with high conceptual complexity in terms of processing and reproducing the information contained in the picture but no specific demand on linguistic complexity, i.e. participants were given no direction on how to structure their language output. Second, we presented a Sentence Generation task with lexical as well as grammatical constraints which was hypothesised to sit in between the Grammar and Narrative tasks in relation to processing cost.

The choice of evaluation parameters for these tasks was derived from the PD literature, with the intent to capture a wide range of language production elements. We therefore investigated the level of grammatical correctness, the complexity of the language produced as well as the information content where relevant. To maintain a reasonable power for the statistical results, the number of variables was restricted to seven per task.

*Complex grammatical structures (henceforth Grammar Tasks)*. As the name suggests, this task was designed to assess complex grammar and focused on the comprehension and production of passives and relative clauses, which have been highlighted as problematic in previous investigations of language problems in PwPD. Executive function deficits have been implicated in reduced performance in such tasks, in particular inhibition, set shifting and working memory [8, 10, 51, 52, 77]. As most of the literature to date has been published on English speakers, there were no assessment materials available for this study that had been validated for the PD population in German. Instead, we used relevant materials from resources for the evaluation and treatment of complex sentence structures in aphasia [78]. In addition, we designed a further task based on the same grammatical structures but with higher complexity to pre-empt a ceiling effect in case these tasks were too easy for our participants. The three tests were always presented in the same order, starting with the comprehension task immediately followed by the production test. We included both comprehension and production in our investigation as comprehension generally results in better performance in healthy controls, and the comparison thus contributed to our assessment of whether the PwPD were disproportionately impacted by task complexity in addition to varying this across the three tasks. It also allowed the participants to familiarise themselves with the sometimes unusual grammatical constructions they had produce subsequently. As we wanted to investigate as wide a range of conditions as possible, we had to reduce the number of tokens to eight per task.

Grammar Task 1 (Simple Relative Clauses, SRC [78]) focused on subject and object relative clauses (SR and OR) involving two agents (Table 2). All relative clauses were right branching, i.e. they followed the object of the main clause. The main clause consisted of a fixed carrier clause in the form of “I see the X…”. Depending on the gender and number of the agent, the nature of the relative clause (subject or object relative) is either signalled through the form of the relative pronoun/determiner, or the form of the verb in German. An equal number of each condition was included in the task.

**Table 2:** Example structures for grammar tasks 1–3.

*Sentence type*	*Example*
**Task 1—Simple Relative Clauses (SRC), 2 agents**	
Subject relatives (SR), n = 4	Ich sehe den **König**, der den **Sohn** misst. (I see the king who measures the son,) Ich sehe das **Rind**, das die **Frauen** schiebt. (I see the bull which pushes the women.) The target agent in the main clause (king/bull) is the subject of the subordinate clause
Object Relatives (OR), n = 4	Ich sehe den **König**, den der **Sohn** misst. (I see the king who the son measures / who is measured by the son.) Ich sehe das **Rind**, das die **Frauen** schieben. (I see the bull which the women are pushing/ is pushed by the women.) The target agent in the main clause (king/bull) is the object of the subordinate clause
**Task 2—Passives (PASS)**	
Active, n = 4	Der **König** misst den **Sohn**. (The king measures the son.)
Passive, n = 4	Der **Sohn** wird vom **König** gemessen. (The son is measured by the king.)
**Task 3—Complex Relative Clauses (CRC), 3 agents**	
**Embedded relative clauses**:	
Subject with Subject Relative (S-SR), n = 2	Der **Hase**, der über den **Hund** springt, jagt die **Maus**. (The rabbit, which jumps over the dog, chases the mouse.) The subject of the main clause (dog) is also the subject of the relative clause
Subject with Object Relative (S-OR), n = 2	Der **Hund**, über den die **Maus** springt, jagt die **Katze**. (The dog, over which the mouse jumps, chases the cat.) The subject of the main clause (dog) is the object of the relative clause
**Right-branching relative clauses**	
Object with Subject Relative (O-SR), n = 2	Die **Maus** jagt den **Hasen**, der über die **Katze** springt. (The mouse chases the rabbit, which jumps over the cat.) The object of the main clause (rabbit) is the subject of the relative clause
Object with Object Relative (O-OR), n = 2	Die **Katze** jagt den **Hasen**, über den die **Maus** springt. (The cat chases the rabbit, over which the mouse jumps.) The object of the main clause (rabbit) is also the object of the relative clause

**Abbreviations**: n = number of stimuli in task, S = subject, O = object, SR = subject relative clause, OR = object relative clause. The agents to be processed have been marked in bold.

Grammar Task 2 (Passives, PASS [78]) investigated active and reversible passive structures (Table 2), gain involving two agents. It was assumed to be less complex than grammar tasks 1, as it contained a simple clause structure with a Subject-Verb-Object (SVO) structure.

Grammar Task 3 (Complex Relative Clauses, CRC, own design) tested subject and object relative clauses involving three agents. For this task, four sentence types were constructed, each including a main and a relative clause, with both the main and relative clauses consisting of Subject-Verb-Object structures. The relative clause was either a subject or object relative clause and either followed the subject (embedded) or the object of the main clause (right branching) (Table 2). The assumption was that the additional agent (increased conceptual complexity), as well the embedded nature of some of the relative clauses (higher linguistic complexity) would make this task more demanding than the SRC task, and thus the most complex of the three grammar tasks. There was further variation of complexity within the task, i.e. embedded relatives were predicted to be more complex than right-branching ones, and object relatives more complex than subject relatives.

All materials (pictures and stimulus recordings) were presented in PowerPoint slides, with participants and the examiner sitting side by side looking at the screen. Participants indicated when they were ready to move on to the next item. Response times between stimulus presentation and response initiation were monitored but did not suggest any noteworthy differences between or within the participant groups. They were therefore not included in the statistical analysis. Participants worked through four example sentences before starting each task. For the comprehension part of the tasks they were presented with two alternative pictures representing the opposing grammatical structures (i.e. subject vs object relative, passive vs active) and had to choose the picture that corresponded to an orally presented stimulus. These stimuli had been pre-recorded by a speaker of standard German to ensure consistency in presentation across participants.

**Fig 1:**
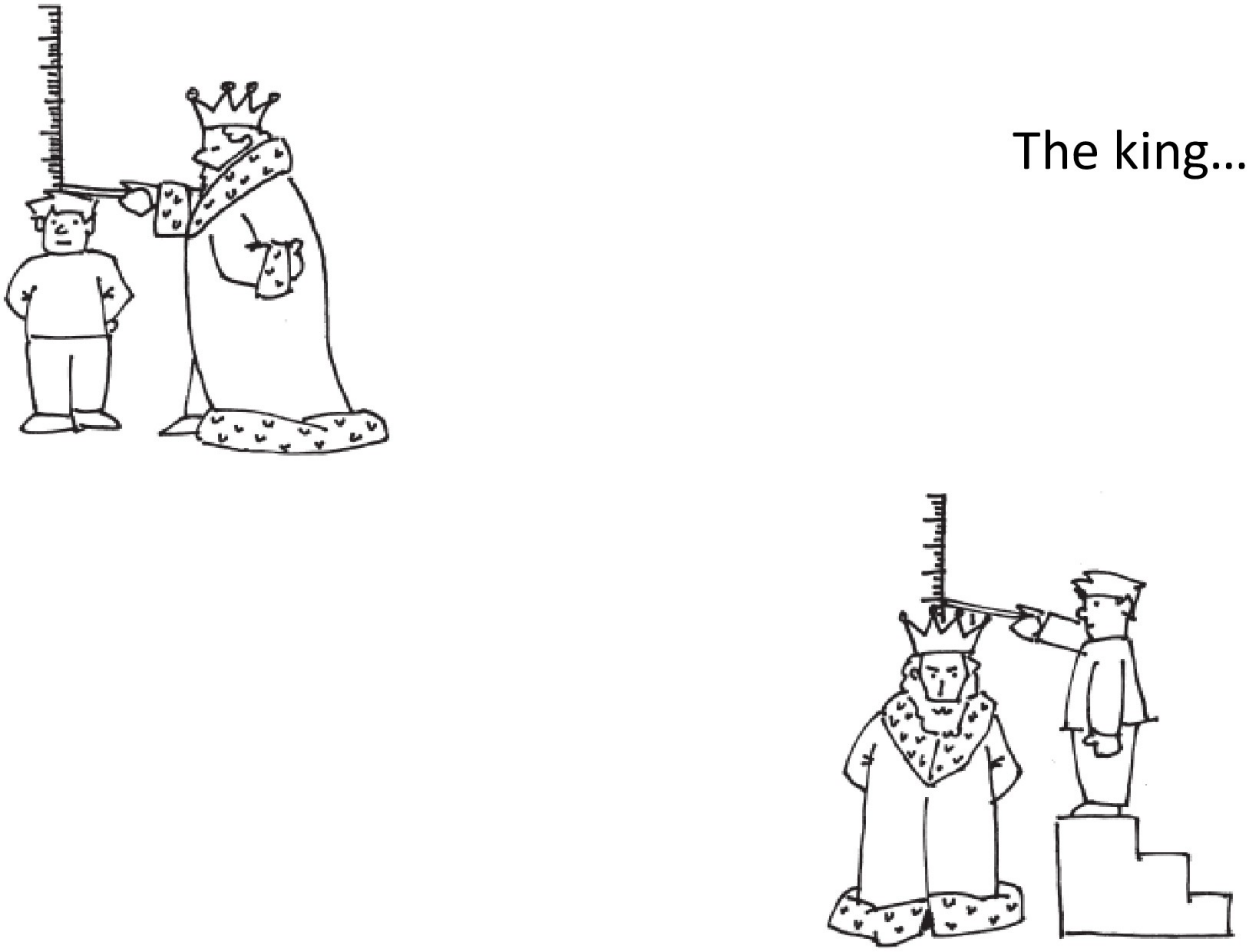
Example of a stimulus slide for the Passives production task (drawings Copyright by NAT-Verlag Hofheim, Germany).

For the production part of Tasks 1 and 2, two pictures relating to the opposing grammatical structures were again presented on the page, in addition to an indication of the start of the sentence (e.g. “I see the king…” (SRC) / “The king…” (Passives), Fig 1). This was to ensure that the correct structure was elicited. The examiner produced the linguistic structure corresponding to one of the pictures (e.g. SRC: “I see the king who measures the son”; Passives: “The king measures the son”), this then prompted the participant to produce the opposite structure, in this case the passive equivalent (SRC: “I see the king who the son measures”; Passives: “The king is measured by the son”).

For Task 3, the participant only saw one picture and had to describe what was happening in it. To elicit the appropriate structure, they were provided with the order in which the agents had to be named in the utterance, e.g. “mouse–rabbit—cat” for the target “The mouse chases the rabbit which jumps over the cat”.

The participants’ performance in the comprehension task was scored as number of items correctly identified. For the production variant, a more detailed 4-point scoring system was devised due to the wider range of responses evident in both groups, as follows:

- 3 points—able to produce the target structure without self-correction or prompting;
- 2 points—able to produce the target structure after self-correction;
- 1 point–able to produce the target structure correctly with help of a prompt;
- 0 points–unable to produce the target structure/ incorrect production despite prompt.

No further analysis of error type was conducted for this task, as these were determined mostly by the task structure, e.g. passive tasks are designed to elicit errors with thematic role assignment, word order or morphology whereas relative clause tasks focus more on the morphology of the relative pronoun or verb. They were thus not easily comparable across the Grammar Tasks or with the more naturalistically generated language in the other tasks described below. Two sets of results were drawn from this analysis—(1) the mean score per task per participant to reflect overall accuracy of performance, and (2) the percentage of false starts (scores of 2) relative to all correct productions of the target utterance (sum of scores of 2 and 3).

*Sentence generation*. This was viewed as a task that sat between the highly structured grammar tasks described above, and the more naturalistic picture description below in terms of complexity. Participants were presented with five noun-verb pairs and asked to produce a “long” sentence. They were provided with a model which included a main and subordinate clause, but were not obliged to do so by the task set-up. Three word pairs consisted of highly related words (prepare–interview, unpack–holiday, slip out–door) and two included unrelated words (correct–sunshine, call–river). Whilst this did not allow us to make any statistical comparisons between these categories, the unrelated words were intended to be more difficult and thus provided greater opportunity to identify difficulties for participants who found the task easy to complete. The word pairs were presented side by side on PowerPoint slides, and the examiner proceeded to the next slide once the participant completed the required sentence and indicated they were happy with their production. The task was intended to mirror the Grammar Tasks by requiring the production of a conceptually more complex sentence structure, but provided the participants with greater choice in terms of linguistic complexity, i.e. whether to use a subordinate clause and what type, or whether to go for a simpler, coordinated structure, or in fact just a longer single clause. At the same time, it introduced additional linguistic and cognitive demands in that they had to formulate a sentence that fitted semantically with the words provided, particularly for the unrelated word pairs. The task thus introduced a higher level of constraint than the Narrative task below which left greater choice of the vocabulary used, the sentence construction, and which features of the story were focused on, etc. The following variables were examined:

- mean length of utterance (Sent_MLU): the mean number of words per sentence;
- correct information units (Sent_CIUs, [79]): percentage of CIUs per total number of words;
- sentence complexity (Sent_complexity): the percentage of subordinate clauses in the sample as a function of all clauses;
- grammatical correctness (Sent_errors): percentage of grammatical and lexical errors per number of words;
- false starts (Sent_false starts): percentage of false starts per number of words;
- pausing (Sent_pause): the ratio between articulation and pause time, lower values reflect a greater amount of pause time—both silent and filled pauses were counted.

In addition, we performed a more detailed analysis of error type, distinguishing between lexical-semantic (referred to as lexical henceforth) and grammatical errors, and for the latter between clause (e.g. missing clause elements ([*Mother] is washing the dishes), phrase (e.g. missing phrase elements ([*the] sink is overflowing; inappropriate selection of grammatical function words such as prepositions, determiners, conjunctions, etc.) and morpheme level errors (e.g. inappropriate case (der nach Kekse[*n] sucht (who is looking for biscuits)); verb agreement (he make[*s] preparations)) in line with methodology developed by Crystal [80]. Lexical errors covered inappropriate choice of content words (e.g. verbs, nouns), or grammatical function words where the error was of a semantic rather than grammatical nature. For example, the wrong choice of present perfect auxiliary (to be vs to have) would be scored as a phrase error whereas an inappropriate selection of linking adverb (when vs. if, before vs after) would be classed as a lexical error. The above are reported as percentage of errors as a function of all words produced. A final category covered errors where utterances did not make sense and this was not due to grammatical errors such as missing or wrong order of elements. As they affected the entire utterance they are reported as a percentage of the total number of utterances produced.

*Narrative production*. The final language task consisted of a description of the “Cookie Theft” picture from the Boston Diagnostic Aphasia Examination [76]. As before, the picture was presented to participants on a PowerPoint slide. While there were some constraints put on participants in terms of the topic they had to talk about, they had free choice in relation to complexity of grammatical structures, vocabulary etc. The same variables as for the Sentence Generation task were investigated for the Narrative, including the CIUs (Narr_CIUs), utterance complexity (Narr_complexity), grammatical correctness (Narr_errors), including the detailed error analysis, false starts (Narr_false starts) and pauses (Narr_pauses). In addition, we examined the number of concepts covered in the story (Narr_concepts, total number of concepts generated in the Narrative), applying the framework developed by Mackenzie et al. [81].

The data for the Narrative and Sentence Generation Tasks were transcribed orthographically by a researcher on the project. These were then cross-checked by the first author who is highly experienced in the transcription and analysis of disordered speech, particularly from PwPD. Segments showing differences in transcription were resolved by further listening, combined with an examination of the acoustic speech signal. Where no agreed transcript could be reached due to poor intelligibility, these segments were excluded from analysis. Depending on the length of the unintelligible period, this could be a single word (e.g. “I can see a [X syllables]”) or the entire utterance (e.g. “I can [longer syllable string]”). Inclusion also depended on the type of analysis, for example, example 1 could still be included in the MLU analysis if it was clear that X consisted of a single word, a grammatical error and CIU analysis could be performed on the intelligible items (which would be the only ones contributing to the total number of analysable words score in this case), but a concept analysis would not be possible. Example 2 could allow an error analysis of the first two intelligible items, but no MLU measure. All participants produced sufficient analysable utterances to include the overall tasks in the analysis. We did not perform inter-rater reliability measure for transcription, as this was consensus based. For variables included in the statistical analysis, agreement was as follows (based on re-analysis of six PwPD): 93% agreement for MLU, 97& for complexity, 97% for error type, 100% for false starts. Inter-rater reliability for the Narrative (four PwPD) was 94% for CIUs and 100% for concepts. All difference could be satisfactorily resolved subsequently and no items had to be removed from the analysis. In addition, data were analysed acoustically to extract pause data. This was done with Praat [82], version 6.0.43) using an automatic script which was then checked manually for accuracy. Pauses were defined as periods of silence with a minimum duration of 200 ms, the average percentage difference between duration measures was 0.10%

### Statistical analysis

Statistical analyses were performed using IBM SPSS Statistics 25 for Windows (2017). For between-group comparisons necessary to answer question 1, data were tested for normal distribution with Shapiro Wilks tests and homogeneity of variances with Levene’s tests, with subsequent use of parametric (t-test) or non-parametric tests (Mann-Whitney-U-test) as appropriate. Due to the exploratory design and the resulting relatively low number of tokens for the Grammar and Sentence Generation Tasks, and the high number of variables assessed, we applied a Bonferroni correction, grouping variables according to language task.

In line with previous studies investigating the relationship between language and cognition, Research Questions 2 and 3 was explored by assessing differences between groups on a single dependent variable (language) after controlling for the effects of one or more covariates (executive function). This allowed us to not only determine whether a relationship existed between the two systems, but also to what degree such a relationship might impact on the difference between PwPD and healthy controls. For this purpose, one-way ANCOVAs were calculated. We used one independent variable with two levels (PwPD vs. healthy controls).

Finally, bivariate correlation analyses were conducted to assess performance relationships across language tasks for Research Question 4, using Spearman’s rho. This was only done for the PwPD.

## Results

### Participant features

As the demographic data demonstrates, the PwPD and control participants were well matched for age, gender and educational level (Table 1). PwPD severity (UPDRS and Hoehn and Yahr score) fell mainly within the mild to moderate continuum, but included some speakers at the more severe range of motor performance. Speech intelligibility only showed mild to moderate impairment, thus facilitating accurate transcription of language performance. Thirteen of 21 participants (PD20 data missing) reported that they experienced problems with language function. The majority (n = 12) highlighted word finding problems, as part of which problems with selecting the appropriate word for the context were reported. The next most common problems were sentence length being reduced and losing the train of thought while speaking, reported by 6 participants for each item. It was unclear from their answers whether reduced sentence length was due to their speech problems or language difficulties. A smaller number of participants (n = 3) indicated they found it difficult to participate in conversations as topics had often moved on by the time they were ready to contribute, and also following complex information. The latter was attributed to loss of concentration though rather than comprehensions difficulties and thus did not reflect an actual language problem.

The statistical comparison between the performance of PwPD and control participants on the cognitive and language tasks highlighted a number of significant differences.

### Executive function

For the cognitive tasks, the Trail Making Contrast (TMT B-A) and letter fluency showed statistically significant differences between the two groups (Table 3).

**Table 3:** Summary of group comparisons for cognitive tasks providing descriptive data as well as statistical results.

Assessment	HC (Mean, SD)	PwPD (Mean, SD)	Test-statistics
PANDA	24.45 (4.92)	22.32 (3.87)	U = 168.00, p = .081
TMT B-A	41.09 (28.10)	98 67 (71.30)	**U = 88.00**, p = **.001**
Digit-span backward	6.68 (2.26)	5.77 (1.97)	U = 179.00, p = .133
BTA	17.91 (2.43)	16.45 (4.68)	U = 205.00, p = .375
Category Fluency	23.27 (5.95)	20.32 (8.14)	U = 183.00, p = .165
Letter Fluency	15.86 (3.63)	12.09 (6.45)	**t(42) = 2.39, p = .021**

**Abbreviations**: PANDA—Parkinson Neuropsychometric Dementia Assessment; TMT B-A—Trail Making Contrast; BTA–Brief Test of Attention

### Language

#### Grammar tasks

Participants completed three different tasks involving passives (PASS), simple relative clauses (SRC) and complex relative clauses (CRC). The level of difficulty was hypothesised to progress from passives being the simplest and complex relative clauses the most difficult task. Comprehension of these structures was assumed to be easier than production. The group comparison of the three tasks (Table 4) indicates that the PD group performed similarly to the control group except during the production of CRC (CRCprod), suggesting that this might have been the most difficult task. However, the descriptive statistics suggest that performance in the CRC task was on the whole better than for SRC.

**Table 4:** Summary of group comparisons for the error rate in the grammar tasks providing descriptive data as well as statistical results. Higher scores indicate better performance. Results significant with Bonferroni corrections are marked in bold.

Grammar Task (Significance level with Bonferroni-correction: .008)			
Measure	HC (Mean, SD)	PwPD (Mean, SD)	Test-statistics
SRCcomp	88.10 (12.50)	81.81 (12.03)	U = 165.50, p = .060
PASScomp	96.59 (8.78)	96.59 (8.78)	U = 242.00, p = 1.00
CRCcomp	93.75 (10.74)	85.23 (15.73)	U = 159.50, p = .035
SRCprod	83.59 (17.56)	69.05 (23.49)	U = 155.00, p = .040
PASSprod	93.95 (8.64)	95.18 (8.69)	U = 224.00, p = .627
CRCprod	89.91 (16.05)	76.09 (19.53)	**U = 98.00, p = .001**

Abbreviations: SRC–Simple Relative Clause; PASS–passive; CRC–Complex Relative Clause; comp–comprehension; prod–production

In addition to error rate, the percentage of false starts in the production tasks was investigated, but there was no significant group difference evident for either of the tasks.

#### Sentence generation and narrative tasks

Contrary to expectation, the PwPD did not show many differences in language performance to the control group (Table 5). The only parameter to show consistent differences across the groups was the number of errors produced. In addition, PwPD showed a higher number of false starts in the Sentence Generation, but not the Narrative task. Although some other measures such as the percentage of pause time and the number of CIUs in the Narrative were suggestive of differences, the statistical results were no longer significant once the Bonferroni correction was applied.

**Table 5:** Summary of group comparisons for the sentence generation and the narrative tasks providing descriptive data as well as statistical results. Results significant with Bonferroni corrections are marked in bold.

Sentence Generation (Significance level with Bonferroni-correction p < .008)			
Measure	HC	PwPD	Group Comparison
Sent_MLU	6.45 (1.02)	6.86 (2.47)	t (41) = -0.703, p = .486
Sent_CIU	99.8 (0.61)	94.66 (10.05	U = 170.00, p = .037
Sent_complexity	32.62 (14.98)	34.09 (17.44)	U = 229.50, p = .971
Sent_errors	0.44 (0.84)	2.01 (2.03)	**U = 120.00, p = .003**
Sent_false starts	1.58 (2.01)	3.59 (3.30)	**U = 75.00, p < .001**
Sent_pause	84.73 (9.76)	76.16 (16.65)	U = 128.50, p = .021
Narrative Production (Significance level with Bonferroni-correction p < .007)			
Measure	HC	PwPD	Group Comparison
Narr_MLU	6.30 (2.14)	5.36 (2.01)	U = 159.50, p = .125
Narr_CIU	86.80 (9.23)	76.71 (14.72)	t(40) = 2.66, p = .011
Narr_Concepts	5.86 (0.85)	5.14 (1.15)	U = 143.50, p = .043
Narr_complexity	34.25 (23.96)	34.18 (27.34)	t(40) = .009, p = .993
Narr_errors	0.83 (0.91)	2.85 (2.60)	**U = 114.50, p = .006**
Narr_false starts	2.03 (2.13)	3.19 (3.02)	U = 169.50, p = .196
Narr_pauses	72.62 (10.49)	62.52 (15.16)	U = 128.50, p = .021

Further descriptive analysis of the error data revealed that the significant group differences were due to a higher number of PwPD producing errors rather than error rates per individual being higher. That is, 72% of PwPD made errors in Sentence Generation compared to only 23% of control participants, and 77% PwPD in the Narrative compared to 46% of controls. This led to statistically significant differences for number of morpheme errors in the Narrative (U = 147.00, p = .004) and lexical errors in Sentence Generation (U = 176.00, p = .016). Lexical errors tended to consist of semantically similar items that were incorrect or inappropriate for the context, e.g. Hochstuhl (high chair) instead of Stuhl (chair), or “der Stuhl faellt herum” (the chair falls about) rather than “der Stuhl faellt um” (the chair falls over). Some confusion with adverbs was also identified, e.g. “wenn” (when) instead of “als” (as) in “er reicht zur Keksdose wenn [als] der Stuhl umfaellt” (he tries to reach the cookie jar when [as] the chair tips over, or “Heute morgen war ich sehr aufgeregt, um mich auf das Vorstellungsgespräch vorzubereiten” (this morning I was very nervous in order to prepare for the interview). Morpheme errors tended to consist of inappropriate use of tense, and wrong case markings on adjectives and determiners. These characteristics were similar across the two tasks.

One error category that only occurred in the PwPD group in the Sentence Generation task were sense errors, resulting in a significant group difference (U = 187.00, p = .034). These reflected errors where it was difficult to tie the various utterance elements into a coherent meaning. In some of these cases, this was due to omission of longer grammatical units or a combination of grammatical, lexical and/or pragmatic errors. For example, in the utterance “Um den Urlaub gut zu gestalten, muss man probepacken, und dann wieder auspacken, wieviel man mitnehmen soll” (in order to plan your holiday well, you have to sample pack and then unpack again how much to take with you) it appears that a subordinate clause has been omitted, such as “…**in order to know** how much to take”. On the other hand, “Es ist mit dem Wetter genau so, rauszuschlüpfen und dann durch die Tür zu gehen um festzustellen, dass es doch nicht so ist” (word pair: slip out–door: "It’s exactly like this with the weather, to slip out and then to go through the door to realise that it’s not like that afterall”) appears to contain issues with wrong word choice and missing reference. Finally, some structures were largely grammatically and semantically correct, but there was no identifiable logical link between the sentence elements, e.g. “(Wenn wir über die) der Fluss verläuft unter der Brücke hindurch, dann (schrei*) rufen wir immer um Hilfe” (word pair: call–river: “(when we over the) the river runs under the bridge, then we always (shout*) call for help”), or “Die ganze Jungs rufen Fluss, weil sie am Wasser spielen” (all the boys call river because they are playing near the water).

We also correlated the self-reports with the error data, both for all types of errors and error totals, as well as for lexical errors only with reports of word finding difficulties. None of the correlations were significant with the exception of lexical errors in the Sentence Generation Task and the self-reports of word finding problems (r = .533, p = .013) as well as total language difficulties (r = .484, p = .026).

### Relationship between language and cognitive function

The group comparison showed significant differences across both cognitive and language components, indicating a possible relationship between these factors. In line with Troche and Altman’s [13] study, we went beyond a pure correlation analysis and instead controlled the linguistic variables that had consistently highlighted group differences (the error rates for all three language tasks) with cognitive parameters, i.e. the executive functions that had highlighted group differences (Trail Making Contrast), as well as the cognitive screening assessment (PANDA). Although Letter Fluency had also indicated significant group differences, we excluded the task from this analysis as it is actually considered a language or hybrid task by some researchers, e.g. [83, 84], and would thus have confused matters. We performed an ANCOVA using group as the fixed factor, the cognitive assessments as the covariate, and the individual language tasks as the dependent variable. For the Trail Making Contrast, results indicated that grammatical errors in Sentence Generation and the Narrative continued to distinguish between the PwPD and HC groups (Sent_errors: *F*(1,39) = 4.87, *p* = .033, *η*_*p*_^*2*^ = .789; Narr_errors: F(1,38) = 10.46, *p* = .003, *η*_*p*_^*2*^ = .175). On the other hand, the performance in the Complex Relative Clause production task was no longer significantly different in the PwPD once the Trail Making Contrast was taken into account (CRCp: *F*(1,40) = 1.13, *p* = .295, *η*_*p*_^*2*^ = .014). Performance in the highly structured Grammar Tasks was thus linked to the participant’s set-shifting ability, whereas error rates in more naturalistic tasks (Sentence Generation and Narrative) appeared to reflect more inherent language deficits in the PwPD.

In terms of the PANDA results, Narrative and Sentence Generation performance remained distinct between groups (Sent_errors: *F*(1,40) = 4.68, *p* = .036, *η*_*p*_^*2*^ = .048; Narr_errors: F(1,39) = 9.10, *p* = .042, *η*_*p*_^*2*^ = .903), but the error rates in the Complex Relative Clause production task now also distinguished between groups (CRCp: *F*(1,41) = 4.23, *p* = .046, *η*_*p*_^*2*^ = .024). This suggests that linguistic error rates in the CRC were more specifically related to the level of executive functioning, in this case set-shifting, than more general cognitive performance as reflected by the PANDA.

### Performance across language tasks

To answer the question how language performance compared across the three task categories within the PD group, we correlated comparable variables across the three language tasks. These included the total number of grammatical errors and number of false starts for all three tasks, and utterance length, utterance complexity, and pausing for Sentence Generation and Narrative. Results confirmed the close relationship between the structured grammar tasks both in terms of error rates and false starts (Table 6). There were also some significant correlations between false start and error rate parameters across the three task types, but no particular pattern was apparent that would allow one task to predict performance in another. The analysis of further measures from the Narrative and Sentence Generation task revealed a significant correlation between articulation/pause time ratio across the two tasks as well as the MLU in the Narrative and pausing in the Sentence Generation task. The remaining correlations were all limited to within task relationships and all for Narrative parameters, i.e. MLU was correlated with complexity and pausing, and complexity with the number of CIUs (Table 6). Besides the pause measures, there was thus no clear relationship between similar parameters across the two tasks. Further descriptive analysis into error rates across the two tasks where a relationship was anticipated given the error analysis above revealed that the lack of a significant correlation was due to the fact that whilst the error rates might have been comparable, different participants produced these errors across the two tasks. The data show that out of the 21 participants who completed the tasks, only just under 50% showed a consistent performance, i.e. one was error free in both tasks, and a further nine produced errors across the two. The remaining eleven PwPD only showed errors either in Sentence Generation (n = 4) or the Narrative (n = 7).

**Table 6:** Results for the correlational analysis of comparable parameters across language tasks.

Comparisons across all tasks			Comparisons between Sentence Generation and Narrative		
Measure 1	Measure2	Spearman’s rho	Measure 1	Measure2	Spearman’s rho
SRCprod	PASSprod	.481, p = .023	Narr_pause	Sent_pause	.638, p = .002
SRCprod	CRCprod	.510, p = .015	Narr_MLU	Sent_pause	.677, p = .001
CRCprod	Sent_false starts	-.523, p = .015	Narr_MLU	Narr_pause	.712, p < .001
SRC_false starts	SRCprod	-.456, p = .033	Narr_MLU	Narr_complexity	.442, p = .045
PASS_false starts	PASSprod	-.924, p < .001	Narr_CIUs	Narr_complexity	-.552, p = .009
CRC_false starts	Narr_errors	-.472, p = .031			

## Discussion

This study aimed to further elucidate the relationship between language and other cognitive functions in PwPD without dementia across different assessment types and complexities to provide guidance for clinical assessment. Our results highlighted a number of group and task differences which will now be considered in relation to the relevant research questions and hypotheses, followed by a discussion of the clinical implications of these findings.

**Question 1 –**Is there a difference between PwPD and healthy control speakers in relation to selected executive function and language tests?

In line with our hypothesis, the results highlighted a number of differences between the participant groups at both cognitive and linguistic level. These were generally comparable with the literature, for example, the PwPD performed significantly worse in the relation to set-shifting (TMTB-A) and letter fluency despite being matched to the healthy control participants for overall cognitive ability [6, 29]. Our results also showed higher levels of grammatical errors across all language tasks, in particular with regard to morpheme production and lexical choice [10, 13, 41, 52, 53]. In contrast to some previous research there were no significant differences detected in the other language variables or executive function measures, although data trends indicated that at least some PwPD showed similar impairments to those previously reported in the literature for pausing [12], false starts [12, 13], CIUs and concepts [41]. Our participants can thus be considered comparable to those of other research reports, albeit at a lower severity of language and executive function impairment.

**Question 2—**Does the relationship between language and cognition differ depending on the nature of the cognitive assessment?

Our data suggest a complex relationship between language performance and cognition which depends on the nature of the assessment. Initial inspection of the results would suggest that the two functions were independent, i.e. similar to Liu et al. [54], and Lewis et al. [56], the PwPD showed reduced accuracy in language production despite being cognitively matched to the control group with the PANDA. However, further investigation highlighted more subtle differences, indicating that evaluation of the relationship on the basis of cognitive screening tests is not sufficient. Our data indicate that specific cognitive domains, in this case set-shifting, can be affected in the absence of global impairment as reflected by the PANDA score, and can impact on specific aspects of language production. Accordingly, the relationship between language and cognition differed according to which cognitive measure was applied, i.e. the production of Complex Relative Clauses was linked to set-shifting ability but not the participants’ overall cognitive profile. The close connection between set-shifting and grammatical skill is in line with previous research which has implicated inhibition, set shifting and working memory in the ability to process and produce complex sentences [8, 10, 51, 52, 77]. Inhibition and set-shifting are important to supress common Subject-Verb-Object (SVO) structures and allow other syntactic forms to be selected, whereas working memory is essential to retain all information until the end to allow appropriate assignment of thematic roles [10, 28]. Although the current PwPD did not show significant differences in working memory, the set-shifting impairment reflected in the Trail Making Contrast could thus explain the reduced performance in this Grammar Task. On the other hand, the fact that the cognitive screening test was not specifically related to performance on this task should not come as a surprise as it contains assessment of skills that have not been associated with language production, such as visuo-spatial processing or short term memory. As discussed above, the main body of evidence against a link between language and cognition comes from studies that only applied cognitive screening tools rather than relevant executive function tests (e.g. [15, 54, 56]). Our results confirm our hypothesis that the choice of cognitive assessment can impact on the outcomes of such research and that detailed assessment of specific executive functions is necessary to fully explore whether language performance is related to cognitive skills or a separate impairment.

From a clinical point of view, Speech and Language Therapists are more likely to perform cognitive screening assessments than specific executive function tests. In view of the above, this may impact on their overall evaluation of the patient and influence their judgement whether the presenting problems would best be addressed through cognitive or language rehabilitation.

Research Question 3: Does the relationship between language and executive function differ depending on the nature and complexity of the language assessment?

Following on from the above result, the relationship between executive function and language ability was only apparent in the most complex, experimental type language task, the CRC. On the other hand, the more self-generated output from the Sentence Generation and Narrative tasks was not linked to any of the global cognitive or executive function measures. As alluded to above, the relationship of executive functions to the CRC performance was predictable given the previous research highlighting inhibition, set shifting and working memory skills as an essential component in producing such complex sentences [8, 10, 51, 52, 77]. Our findings are also comparable to those of Troche and Altman [13] who report performance differences across their two relative clause tasks, although in their case both PwPD and control groups showed similar performance patterns with no disproportionate impact of complexity on the PwPD. However, in contrast to other research, we did not establish any links between executive functions and the self-generated tasks. This could have been due to the relatively small differences between the PwPD and control speakers. Most of the parameters we investigated did not show significant differences, and although there were group differences with regard to error rate, the number of errors produced in each task was low per individual. This suggests that PwPD were still relatively capable to execute these tasks, and thus did not demonstrate the previously reported relationship to executive function. Our results thus confirm our hypothesis that the more complex, experimental type grammar tasks were more likely to reveal this relationship than self-generated task that allowed the speaker more choice in the linguistic as well as conceptual complexity of their output, at least in our group of participants with no or very mild cognitive impairment.

Clinically, therapists favour structured tests such as the Grammar Tasks that can highlight impairments reliably without the need for time-consuming transcription of speech output and potentially subjective analysis of the results. Whilst our results suggest that such test may confirm the presence of executive function deficits, which in turn favours cognitive rehabilitation as the most effective therapeutic approach, the lack of a relationship between executive function and the self-generated task paradigms is concerning. Our results do not allow us to conclude whether this was due to low levels of severity in our participant group or the fact that these tasks are governed by wider factors, including purely linguistic deficits which would necessitate a different rehabilitative approach. Further research is thus necessary across a wider spectrum of participant characteristics to inform effective intervention for the observed deficits.

**Question 4—**Can the severity and type of impairment in one language task predict those in others?

Our final aim was to investigate whether performance in one language task could predict that in others. The first observation to note in this context is that all three tasks elicited significant differences between the speakers groups and thus appeared equally suited to highlight language difficulties in PwPD. A further positive sign was that there was some consistency regarding which areas were impaired as the PwPD were found to produce a higher rate of grammatical errors across all tasks. It thus appeared that our hypothesis that performance would differ between tasks was not supported by our data. However, this assumption was subsequently disproven both by the additional statistical analysis and the more detailed qualitative investigation of the language output. In other words, there was no meaningful relationship between any of the language measures included in the correlational analysis across the tasks, except for error rate between CRC and SRC which investigated the same grammatical structures, and for pauses between Sentence Generation and the Narrative. This means that impairment measures in the Grammar Tasks could not predict the performance in Sentence Generation or the Narrative. The lack of correlation for grammaticality score can be explained by a closer look at the error profile and language structures generated. Differences between the Grammar Tasks and the other two methods were expected to some degree as the former investigated highly complex structures that are not that common in everyday language, and they were designed to elicit specific types of errors such as verb morphology and word order in the Passive, and relative pronoun and word order in the relative clause tasks. At the same time, all lexical items were provided, sentence length was pre-determined, etc. On the other hand, Sentence Generation and the Narrative required participants to generate information at all levels of language production, thus allowing a wider range of errors. And although the Sentence Generation task required the production of a complex sentence, thus at least partially mirroring the Grammar Tasks, the subordinate clauses produced were mostly temporal (when/before) or causal (because) adverbial constructions rather than the relative clauses included in the Grammar Tasks. Where the latter were produced, these were almost exclusively non-embedded subject relatives which are known to be easier to produce. Sentence Generation thus yielded outputs of lower linguistic complexity than the Grammar Tasks which could explain the difference in profile. In addition, whilst the Narrative and Sentence Generation tasks showed similar type and quantity of errors across the tasks, further analysis suggested that around 50% of PwPD only produced errors in one of these tasks. This means that either both types of tasks should be included to ensure any impairments are picked up, or more data should be collected from one of the tasks to increase the chances of catching such issues. Further research with more impaired speakers is necessary to be able to guide future assessments in this regard.

One error type only apparent in the Sentence Generation task was where the utterance did not make sense. Although some of these could be attributed to more severe cases of the grammatical problems as identified elsewhere in the tasks, others appeared to be more related to problems at the conceptual level. The fact that no similar errors were produced in the Narrative suggests that may have been specifically induced by the constraints imposed by this task, i.e. the level of linguist complexity combined with the need to use specific lexical items. Further investigation with a higher token number and more control of the relationship between the two words presented would be valuable to identify whether this task could be particularly suited to highlight both grammatical as well as conceptual impairments.

In summary, our results indicate that tightly controlled, highly complex language tasks are more likely to show a link between language and cognition than more naturalistic, self-directed tasks. Our data thus support the hypothesis that at least some components of language performance are linked to executive function, as suggested by the majority of the literature [8–10, 12–14, 41], but at the same time highlight that the detection of this link is task dependent. In other words, language tasks that require the comprehension and production of highly complex linguistics structures might not be that different from controlled assessments of cognitive function, i.e. they create artificial speaking situations, include infrequent constructions and have all cues or compensatory strategies removed that would normally be available to the speaker. Performance in such task is thus highly dependent on the executive functions necessary to manipulate linguist structures, such as working memory, set-shifting, inhibition, etc. Even though the same executive skills have been associated with higher order, message level process in language production [12, 13, 53], more naturalistic tasks might require a wider arsenal of cognitive as well as specific linguistic skills and are thus less likely to demonstrate a link to individual executive function tests. It is thus important for future research to investigate language impairment in PwPD across a more diverse set of language tasks to understand this relationship in more depth. In addition, other factors that could impact on language performance, such as task constraints, should be investigated further, particularly in relation to whether they facilitate language production or introduce barriers. Such research should also be able to shed further light on why performance could not be predicted between language tasks when they were supposedly relying on similar underlying cognitive bases. Our results also demonstrate that future studies need to assess cognitive performance in sufficient depth rather than relying on cognitive screening data only as we detected a relationship between cognition and language for specific executive functions (set-shifting), but not for overall cognitive performance (PANDA). In addition, we found that the same group of PwPD can perform similarly to healthy controls on some commonly used task paradigms such as passives and certain types of relative clauses, but be disproportionately impacted by higher levels of complexity. This raises the question whether previous studies that reported no language deficits in the past applied sufficiently complex elicitation tasks.

Our findings have implications for the management of language impairment PwPD, both in relation to assessment and intervention. Our research has demonstrated the importance of conducting detailed assessment of executive function as opposed to cognitive screening and of pitching language tasks at the right level of complexity in order not to miss potential impairments. Further consideration needs to be given to the nature of the assessment task. Due to time restrictions and to increase the reliability of outcomes, clinical language assessments tend to consist of highly structured tasks. Our study suggests that the results gained from such standardised language tests might not necessarily reflect the PwPD’s performance in everyday communication situations. Whilst structured assessments are useful to explore in more detail why a person might be struggling in spontaneous speech situations, language performance in self-generated tasks thus needs to be evaluated to make informed, person centred treatment decisions. In fact, such tasks should form the basis for deciding which structured tests to apply. Self-reports of our participants tended to centre around issues with word retrieval as having the most impact on communication rather than the problems with clause structure, past tense markers or relative clauses identified in our tasks, which impacted on the accuracy but not so much the content of the message. In taking a person-centred rehabilitation approach, a further exploration of the nature, cause and impact of these problems would thus be more appropriate for intervention planning than assessing performance in complex grammatical test conditions. In addition, there was some preliminary evidence self-reported issues can correlated with language assessment outcomes, and this issue requires further investigation in terms of how these can feed into comprehensive assessment of PwPD.

In terms of intervention, our study has confirmed that at least some language deficits associated with PD are closely linked to executive function, in this case set-shifting. There is thus hope that cognitive interventions addressing such issues might have secondary benefits for language production. Unfortunately, with the exception of Altman et al. [60], clinical trials to date have focused insufficiently on language outcomes, largely restricting themselves to verbal fluency and occasionally naming ability. Further research is necessary to identify more clearly how wider issues such as information content, grammatical skills as well as communicative participation can be addressed in future.

Like many other investigations our study suffered from a range of limitations. Whilst all participants performed above the dementia cut-off point for the PANDA [66] and were well matched for cognition, both groups included a wide range of cognitive skills. In particular, four participants in each group could be considered to have mild cognitive impairment (MCI). This number was too small to compare against the cognitively healthy groups, however, further differences in language performance across MCI and healthy groups should be a focus of future research. In addition, the results of this exploratory study need to be confirmed on the basis of a larger participant group and higher token numbers for some of the language tasks to improve statistical power. The breadth of assessment tasks could be widened in future research, whilst we focused on those most implicated in previous research, the scope of assessment should be broadened to gather a fuller picture of the impact of executive function on different language tasks and complexities. A further issue that could not be considered in this study but might be built into future investigations is the fact that cognition can be affected by L-DOPA medication [85]. It would thus be interesting to assess to what degree the observed effects might differ across on and off medication conditions. Finally, whilst we attempted to include a broad range of task types into our experiment, we appreciate that our Narrative task was not comparable to natural language performance. We also restricted our analysis to mainly lexico-grammatical features without consideration of pragmatic components such as turn-taking, variations in language output according to the interlocutors’ level of shared knowledge, understanding of non-literal language, etc., all of which would be important for everyday functioning and thus need further investigation.

In conclusion, this study has provided further evidence for the presence of language impairment in PwPD without dementia across a range of task complexities in line with the previous literature. It identified a link between language and other cognitive aspects, but only for highly complex structures. Whilst the current results need to be confirmed with higher participant numbers they suggest that the diversity in previous findings with regard to the cognitive-language relationship is task related, which also has important implications for clinical assessment.

In order to move forward and apply our research for the benefit of PwPD, we propose that future research focus more closely on the language deficits that have been identified to date and how these affect PwPD functionally. In addition, whilst there remain many unknowns about the nature of language impairment in PD, there now exists a sufficient knowledge base to develop intervention techniques. There are many studies currently underway investigating how cognitive performance can be improved in PwPD, which, similar to Altmann et al. [60], should include functional language variables beyond verbal fluency as outcome measures to assess the treatment’s potential to address these deficits as well. In addition, despite the fact that most of the literature points towards an underlying cognitive deficit as a cause of language impairment, the role of direct language intervention should not be ignored particularly in more naturalistic speaking situations. Whilst cognitive training might improve e.g. lexical retrieval ability globally, furnishing PwPD with a set of strategies on how to deal with e.g word finding problems during conversation could be equally important from a functional point of view.
